# Editorial: Discovery of small molecule lead compounds: A driving force to unravel new anticancer targets and mechanisms

**DOI:** 10.3389/fonc.2023.1117660

**Published:** 2023-02-22

**Authors:** Yan Zhang, Leilei Fu, Muhammad Umer Farooq Awan

**Affiliations:** ^1^ Key Laboratory of Computational Chemistry-Based Natural Antitumor Drug Research & Development, Liaoning Province, School of Traditional Chinese Materia Medica, Shenyang Pharmaceutical University, Shenyang, China; ^2^ School of Life Science and Engineering, Southwest Jiaotong University, Chengdu, China; ^3^ Department of Botany, Government College University, Lahore, Pakistan

**Keywords:** anticancer natural products, leading compound, cancer case analysis, potential cancer targets, target recognition technology

Natural products are complex and rich in structure and have extensive biological activities, which are important sources of new drug creation. They also provide an effective way to search for anticancer lead compounds with enzymes, receptors and genes as new molecular targets, and thus a way to find new anticancer drugs. The target study of natural products is very important to elucidate the mechanism of drug action and the research and development of new drugs. This Research Topic aimed to explore recent developments in this area; seven articles were included. As editors of this Research Topic, it was our pleasure to review a wide range of research articles and reviews within the field. In this editorial, we summarize the main findings and perspectives detailed within each of the accepted articles.

Zhao et al. designed and synthesized a series of bifunctional small molecule inhibitors that bind pharmacophore into the structural framework. Subsequent experiments verified the inhibitory activity of these synthetic compounds against two targets and found compounds with significant pharmacological activity. It suggests that the dual-target drug design approach may provide a new and effective strategy for the discovery of antitumor drugs in the future.

Liu et al. conducted a retrospective case-control study in which 560 eligible patients were selected for continuous enrollment analysis. The relationship between clinical factors, pathological factors, hematologic factors and lymph node metastasis in these cases was discussed. The results of the analysis suggest that surgery for patients with early invasive breast cancer should be more personalized and precise. Appropriate axillary management should be performed on patients who meet the relevant predictors. In another paper of this Research Topic entitled “*Prediction of axillary lymph node pathological complete response to neoadjuvant therapy using nomogram and machine learning methods*”, Zhou et al. conducted a retrospective case study involving 247 patients with early breast cancer (eBC) receiving neoadjuvant therapy (NAT). Pycharm software and five-fold cross-validation analysis were used during the study, and random forest (RF) was used to train and validate the data. The experimental results showed that the advantages of a nomogram are that it is simple, practical and maneuverable. Moreover, machine learning can use clinicopathological information from patients. These predictive models can help surgeons to choose a more rational axillary surgical strategy. These two articles retrospectively analyzed the clinical treatment cases of breast cancer. The treatment strategy and timing of patients were studied, and the nodes of interventional therapy were optimized. These results have extensive reference significance for the better clinical treatment of breast cancer and certain reference significance for the development of breast cancer drugs.

Zhan et al. constructed an “osw-1-target-glioma” cross-network and a “protein–protein interaction network” composed of 151 cross-genes, which predicted ten core targets through the network construction. At the same time, the results of the network prediction were verified by experiments, and OSW-1 was found to be a promising antiglioma chemotherapy drug. One biological activity of natural products often corresponds to multiple targets. It has become an important process in the research of natural anticancer drugs to clarify the target and the mechanism of action. This experiment used the network prediction strategy to predict and verify the target, which is an effective means to clarify the multitarget mechanism of natural products.

Xu et al. studied the effect of irisin on the proliferation of GBM cells and found that it can inhibit the cell cycle of the G2/M phase of GBM cells, induce cell apoptosis and inhibit cell migration. During *in vivo* experiments, irisin was also found to inactivate YAP and inhibit tumor growth in GBM-xenografted mice. These studies suggest that irisin can play an anticancer role in GBM by inhibiting the YAP/β-catenin signaling pathway, thus providing a new strategy for the treatment of GBM.

Microtubules are a very important target for cancer therapy. As a basic component of the cytoskeleton, microtubules are closely related to cell proliferation. Therefore, microtubule stabilizers have become some of the main clinical drugs in the treatment of cancer in recent decades. Taccalonolides—a high oxygen pentacyclic steroidal compound isolated from Taka—are a new kind of microtubule stabilizer. They have similar microtubule-stabilizing activity to the well-known drug paclitaxel and have been shown to reverse multidrug resistance of paclitaxel and epothilone in *in vivo* and *in vitro* model studies. Li et al. systematically reviewed taccalonolides and summarized their structural diversity, semisynthesis, modification and pharmacological activity, providing a new idea for the discovery of microtubule-stabilized drugs.

In the current studies, natural products are often found to have unique anticancer activity, but also the significant advantages of high efficiency, low toxicity and low side effects. Therefore, more and more natural anticancer drugs have been used clinically in recent years. Nan et al. reviewed the anticancer mechanism of natural products. The main death pathway of natural products is apoptosis and immunogenic cell death. At the same time, they also affect the expression of immune cells in the tumor microenvironment, regulate the inflammatory environment and related pathways and have certain therapeutic, intervention and regulatory effects on tumor metastasis.

## Author contributions

YZ conceptualized the Research Topic and was responsible for writing the whole passage. LF and MA edited the manuscript. All authors contributed to the article and approved the submitted version.

